# Reemergence of yellow fever virus in southeastern Brazil, 2017–2018: What sparked the spread?

**DOI:** 10.1371/journal.pntd.0010133

**Published:** 2022-02-07

**Authors:** Joelle I. Rosser, Karin Nielsen-Saines, Eduardo Saad, Trevon Fuller

**Affiliations:** 1 Stanford University School of Medicine, Stanford, California, United States of America; 2 David Geffen UCLA School of Medicine, Los Angeles, California, United States of America; 3 New York City Department of Health and Mental Hygiene, New York City, New York, United States of America; 4 University of California, Institute of the Environment and Sustainability, Los Angeles, California, United States of America; 5 Fundação Oswaldo Cruz, Rio de Janeiro, Brazil; Centers for Disease Control and Prevention, Puerto Rico, UNITED STATES

## Abstract

**Background:**

The 2017–2018 yellow fever virus (YFV) outbreak in southeastern Brazil marked a reemergence of YFV in urban states that had been YFV-free for nearly a century. Unlike earlier urban YFV transmission, this epidemic was driven by forest mosquitoes. The objective of this study was to evaluate environmental drivers of this outbreak.

**Methodology/Principal findings:**

Using surveillance data from the Brazilian Ministry of Health on human and non-human primate (NHP) cases of YFV, we traced the spatiotemporal progression of the outbreak. We then assessed the epidemic timing in relation to drought using a monthly Standardized Precipitation Evapotranspiration Index (SPEI) and evaluated demographic risk factors for rural or outdoor exposure amongst YFV cases. Finally, we developed a mechanistic framework to map the relationship between drought and YFV. Both human and NHP cases were first identified in a hot, dry, rural area in northern Minas Gerais before spreading southeast into the more cool, wet urban states. Outbreaks coincided with drought in all four southeastern states of Brazil and an extreme drought in Minas Gerais. Confirmed YFV cases had an increased odds of being male (OR 2.6; 95% CI 2.2–3.0), working age (OR: 1.8; 95% CI: 1.5–2.1), and reporting any recent travel (OR: 2.8; 95% CI: 2.3–3.3). Based on this data as well as mosquito and non-human primate biology, we created the “Mono-DrY” mechanistic framework showing how an unusual drought in this region could have amplified YFV transmission at the rural-urban interface and sparked the spread of this epidemic.

**Conclusions/Significance:**

The 2017–2018 YFV epidemic in Brazil originated in hot, dry rural areas of Minas Gerais before expanding south into urban centers. An unusually severe drought in this region may have created environmental pressures that sparked the reemergence of YFV in Brazil’s southeastern cities.

## Author summary

In 2017–2018, cities in southeastern Brazil experienced an unusual outbreak of yellow fever virus. In the early 20^th^ century, these cities had large outbreaks of yellow fever, spread by *Aedes* mosquitoes. But until this recent outbreak, they had been free of yellow fever for nearly a century. While this outbreak was spread by *Haemagogus* forest mosquitoes, the reemergence of yellow fever in densely populated urban areas raises serious concerns about it reestablishing ongoing transmission in cities, spread by urban *Aedes* mosquitoes. Our study sought to understand how and why yellow fever virus remerged in this area. We traced the outbreak, finding that it started in hot, dry, rural areas and spread south into cool, wet urban areas. The epidemic coincided with a severe drought, particularly in Minas Gerais where the epidemic started. Individuals with outdoor or rural risk factors were at highest risk, especially when the epidemic started. Therefore, this severe drought may have promoted the spread of yellow fever at rural-urban boundaries. To further explore this idea, we developed a unique framework based on forest mosquito and Howler monkey biology. Our framework, “Mono-DrY,” shows how severe drought could have increased mosquito and monkey densities, promoting the spread of yellow fever.

## Introduction

Between December 2016 and July 2019, a yellow fever virus (YFV) outbreak occurred in southeastern Brazil, resulting in over 2,000 confirmed human cases [1,2]. The height of this epidemic occurred during the summer seasons of 2017 and 2018. This outbreak was unusual both in its large size and geographic distribution. Urban transmission cycles involving *Aedes sp*. mosquitoes were responsible for severe YFV epidemics up until the early 20^th^ century, when aggressive vector-control and vaccination campaigns eliminated this type of transmission pathway [3]. However, since the early 1940’s, YFV in Brazil has circulated exclusively by a sylvatic transmission cycle in which *Haemagogus sp*. mosquitoes, which live and breed in forest canopies, feed primarily on non-human primates (NHPs) and only sporadically feed on humans [4–6]. This results in low human transmission levels with periodic small outbreaks occurring in rural northern and western forest states of Brazil.

In contrast, the 2017–2018 epidemic occurred in southeastern Brazil, including São Paulo, Espírito Santo, and Rio de Janeiro, urban states which had not experienced YFV transmission in nearly a century. Outbreaks of YFV in these urban states raise great concern for the possibility of an urban transmission cycle resurgence involving *Aedes aegypti* and *Aedes albopictus* [7,8]. These two mosquitoes preferentially breed in small water containers and therefore thrive in urban slums with poor water infrastructure [9,10]. In recent decades, Espírito Santo, São Paulo, and Rio de Janeiro have experienced several large outbreaks of other flaviviruses such as dengue, chikungunya, and Zika which are transmitted by these *Aedes* mosquitoes [11–13]. If YFV reestablished an urban cycle involving *Aedes* in these densely populated, inadequately vaccinated states, the public health impact could be enormous.

Curiously, albeit fortunately, there has been no evidence of *A*. *aegypti* infection in the 2017–2018 YFV epidemic [8,14]. This in no way precludes the possibility of a future spillover to *Aedes aegypti*. However, this particular epidemic was driven by conditions promoting transmission by *Haemagogus* mosquitoes [14]. This epidemic started in Minas Gerais, a rural state uniquely positioned in a transition zone between forested areas to the northwest and coastal states in the southeast, which had previously experienced only sporadic YFV transmission [1,15]. The massive outbreak of YFV in Minas Gerais and unprecedented incursion through southeastern Brazil in 2017, driven by forest mosquitoes, suggests that a change in environmental conditions triggered a new transmission dynamic in this region. Notably, this epidemic coincided with one of the most severe droughts this area had experienced in the past century [16–18].

A number of environmental, viral, and human factors have been identified contributing to the evolution of this epidemic [19–23]. Yet the question remains why this epidemic happened at this particular time, and place, and with these forest dwelling mosquitoes. Furthermore, whether and how this drought could have triggered this epidemic has not been previously scrutinized. Better understanding of why this epidemic occurred can help us predict and prepare for future outbreaks.

In this study we explore the environmental conditions at the origin of the epidemic and the potential biological mechanisms that may have triggered this unusual outbreak. We hypothesize that a severe drought during this time set the stage for YFV transmission to intensify at the rural-urban interface and then spread into urban areas. To assess this hypothesis, we traced the evolution of human and non-human primate (NHP) cases to reconstruct the spatiotemporal origins of this epidemic. We examined the timing of the epidemic waves in each state in relationship to a drought index. We analyzed a database on individual YFV cases during this epidemic to evaluate demographic characteristics that are reasonable proxies for rural and outdoor exposure to further examine where and how YFV likely entered the human population. Finally, we created a biologically plausible mechanistic framework to demonstrate how drought could have sparked the spread of this epidemic.

## Methods

### Geographic distribution of human and NHP cases over time

The number of confirmed human YFV cases by municipality were obtained from the Sistema de Informação de Agravos de Notificação (SINAN), the Brazilian national health surveillance system [24]. Suspected cases of YFV were classified as confirmed in the SINAN database based on whether the case had serologic or polymerase chain reaction evidence of an acute YFV infection. Southeastern Brazil experiences two main seasons: the more cool and dry ‘winter’ season from May to October and the more hot and wet ‘summer’ season from November to April, with peak rainfall and high temperatures in January to February. Based on reported date of symptom onset and seasonal changes, cases were divided into time periods to show the progression of case counts over the course of the epidemic.

We also obtained data from SINAN on the number of non-human primates (NHPs) that were found dead, tested positive for YFV, and were reported by state health departments to the Division of Zoonoses and Vector-borne Diseases of the Ministry of Health from January 2007 to December 2020 (data received from SINAN) [24]. The surveillance included five genera of NHPs: Alouatta, known as howler monkeys which are large diurnal NHPs that forage at the tops of canopies and are adapted to degraded environments, Callithrix, which are small marmosets whose diet consists of insects, fruits and other plants, and Cebus, Saimiri, and Sapajus, all of which are medium-sized NHPs that live in large troops [2]. We pooled the genera and created a map of the municipalities reporting at least one confirmed NHP case in each of 2016, 2017, and 2018.

Human and NHP cases were mapped to municipality in R Studio version 1.1.456 using the geobr package [25] which pulls municipality boundaries from the Instituto Brasileiro de Geografia e Estatistica (IBGE) [26].

### Geographic distribution of temperature, rainfall, and population density across southeastern states of Brazil

Raster data for the historical (1970–2000) monthly mean, minimum, and maximum temperature and mean rainfall was obtained from WorldClim [27], and then mapped for the southeastern states of Brazil to depict averages during the summer season (November to April) and winter season (May to October) over this geographic region, using R Studio and the geobr package [25]. Data on population density in 2017 across this region was obtained from WorldPop and mapped in R Studio [28].

### Drought

To assess drought conditions, we obtained precipitation and temperature for each month from January 2007 to December 2020 from weather stations in each state. We queried the databases of the Brazilian National Institute of Meteorology and National Water Agency for all stations in each state and selected the weather station nearest to the municipality with the greatest number of human cases of YFV. If two stations were equidistant from the municipality, we selected the one with the fewest missing observations during the study period. This resulted in the selection of weather stations in Vitória, Espírito Santo; São Paulo, São Paulo; Teófilo Otoni, Minas Gerais; and Campos dos Goytacazes, Rio de Janeiro.

These data were used to calculate the monthly Standardized Precipitation Evapotranspiration Index (SPEI), a well-established drought index which incorporates temperature and the evapotranspiration potential into the commonly used Standardized Precipitation Index (SPI) [17]. A negative value of the index indicates that the evapotranspiration exceeded precipitation, resulting in a water deficit. The SPEI classification of drought is as follows: ≤ -2.0 is extremely dry, -1.99 to -1.5 is severely dry, -1.49 to -1.0 is moderately dry, -0.99 to 0.99 is near normal, 1.0 to 1.49 is moderately wet, 1.5 to 1.99 is severely wet, and ≥ 2.0 is extremely wet. Months in which the SPEI assumed a negative value were anomalously hot and dry, compared to the average climatic conditions during the study period. We defined drought as months in which the SPEI was less than zero. To calculate the index, we utilized the aforementioned weather station data, and the R package SPEI 1.7. The SPEI settings consisted of a scale parameter of 8 months and a Gaussian kernel.

### Rural/Outdoor exposure

Rural or outdoor exposure was assessed based on relevant demographic characteristics including sex, working age, recent travel history, and occupation as reported to SINAN in 2017 and 2018 [24]. Working age was defined as being between the ages of 16 and 65 years old, the minimum legal working age and the retirement age for men in Brazil, respectively. Municipal Health Departments use a standardized Case Report Form to report YFV cases to SINAN. This form indicates the patient’s municipality of residence as well as the municipality where the patient was believed to have been infected within the two previous weeks. We classified a patient as having traveled if the municipality of residence was different from that of infection. To categorize municipalities as rural or urban, we utilized the typology of the Brazilian Institute of Geography and Statistics [29]. This typology is based upon population density and the extent of built-up areas according to satellite images. We then divided travel history into five categories based on the direction of travel between rural and urban areas.

Cases were classified in the SINAN databased as confirmed or discarded based on a Ministry of Health review of the individual laboratory and clinical case presentation; inconclusive cases were not included in the analysis. Demographic risk factors for being a confirmed versus discarded case were evaluated with univariate and multivariable logistic regression. YFV vaccination status was evaluated and controlled for in the multivariable logistic regression. Month and year were also included in the multivariable regression to control for seasonal travel trends.

For the subset of confirmed cases for whom occupation was recorded, occupation was classified into one of four categories: farmer/rural worker, other outdoor worker, indoor worker, or ‘intermediate’ (for jobs which could be both indoors and outdoors). To assess whether rural/outdoor occupational risk was higher at the beginning of the epidemic, the number of confirmed cases with a known occupation of farmer/rural worker versus other occupations were compared between the first (2017) and second (2018) years of the epidemic and between the start of each new wave (January) and subsequent months (February through December) using Fisher’s exact test.

The human data used in this analysis was deidentified data available from an existing public access database (SINAN) so human subjects ethical review was not required.

## Results

### Geographic distribution of human and NHP cases over time

**Fig 1:**
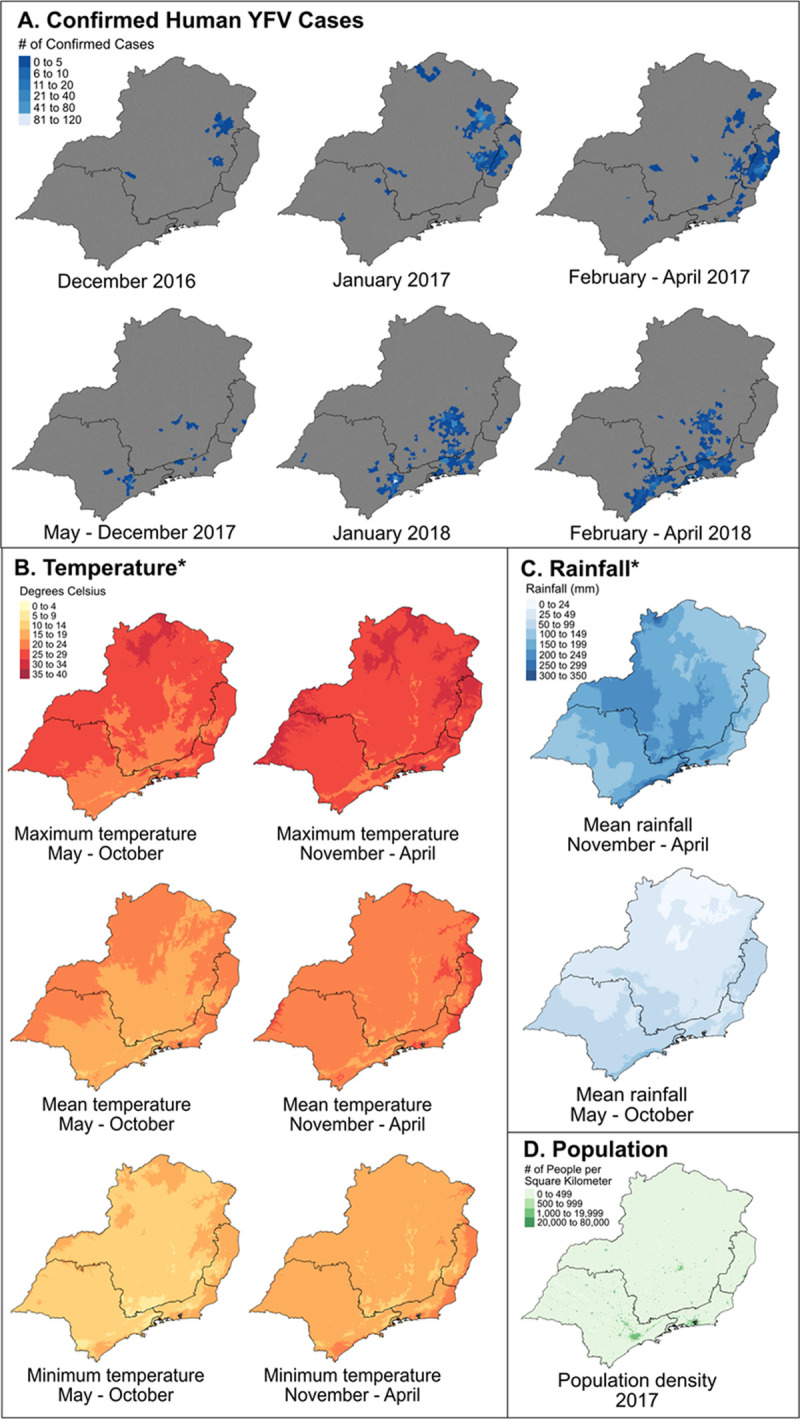
Yellow fever virus outbreak emergence in hot, dry rural areas of southeastern Brazil. Abbreviations: YFV = yellow fever virus; mm = millimeters. A. The YFV outbreak was first identified in the northeastern region of Minas Gerais and subsequently spread east into EspÌrito Santo and then south into São Paulo and Rio de Janeiro. B. The northeastern region of Minas Gerais and EspÌrito Santo experiences the highest temperatures in this region. Maps on the left show the cooler winter months and on the right show the warmer summer months. C. The northern area of Minas Gerais is also the driest part of this region. D. The northeastern region of Minas Gerais is also the most rural and least densely populated part of this region. YFV case data received from SINAN [24]. Municipality boundaries available from the Instituto Brasileiro de Geografia e Estatistica (IBGE). (https://portaldemapas.ibge.gov.br/portal.php#homepage) [26]. *Temperature and rainfall values are derived from the historical average (1970–2000) from WorldClim [20].

**Fig 2:**
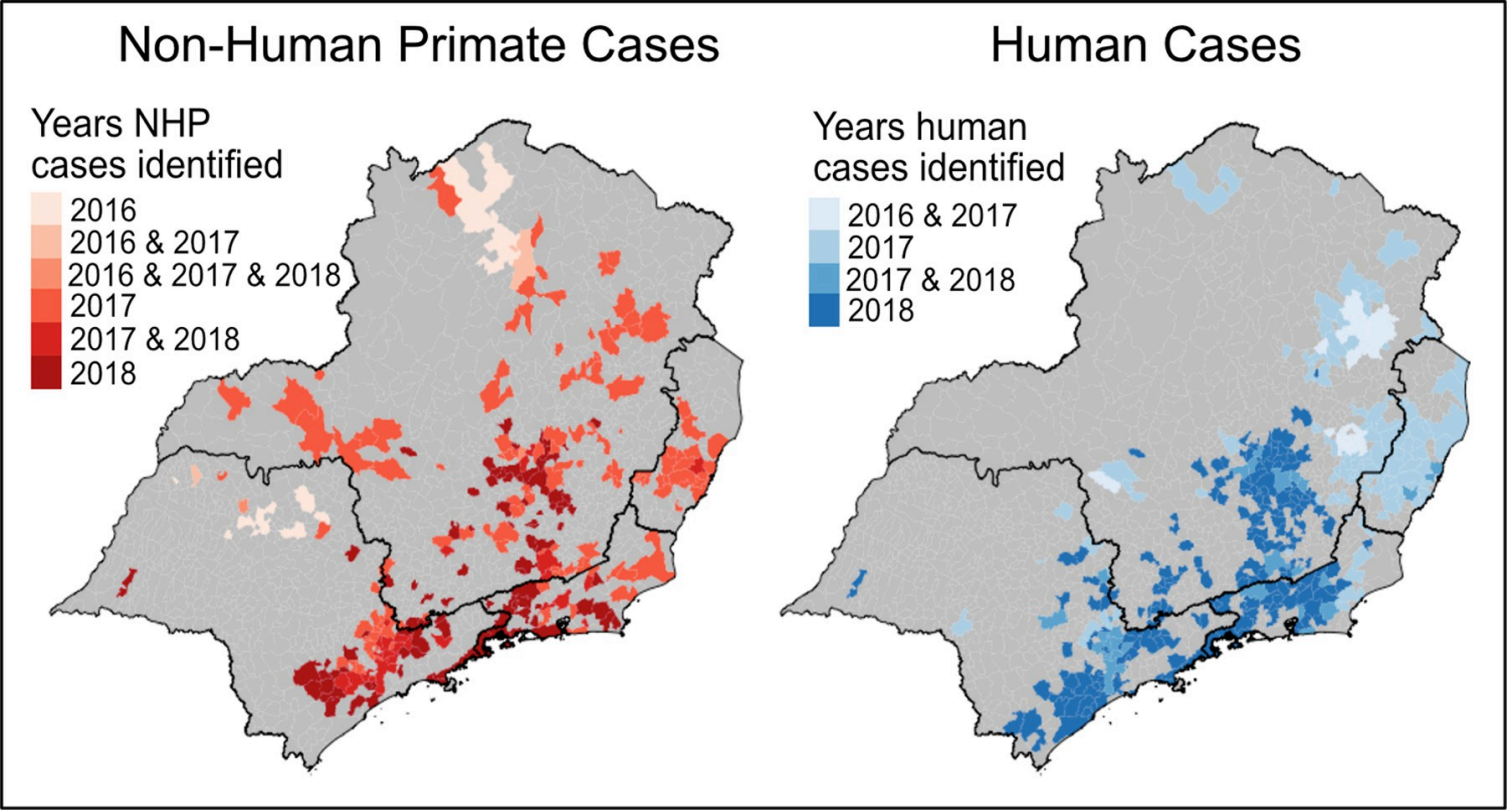
NHP & human yellow fever virus cases by year. Abbreviations: NHP = Non-human primate. NHP cases (left map) were identified in the rural, northern area of Minas Gerais starting in 2016 and continuing through the epidemic in 2017 and 2018. The earliest human cases (right map) identified during this outbreak also occurred in northern Minas Gerais in 2016 and 2017. As the epidemic progressed through 2017 and 2018, both NHP and human cases spread south into the urban centers of Rio de Janeiro and São Paulo. YFV case data received from SINAN [24]. Municipality boundaries available from Instituto Brasileiro de Geografia e Estatistica (IBGE) (https://portaldemapas.ibge.gov.br/portal.php#homepage) [26].

The first human cases of YFV in 2016 were identified in December in the northeastern region of Minas Gerais, with case counts increasing in intensity and spreading from that initial epicenter towards the border of Minas Gerais and Espírito Santo in January 2017, and later spreading south into Rio de Janeiro and São Paulo. (Fig 1) The geographic progression of cases follows a climactic trajectory, starting in the hot, dry northern part of the region and steadily progressing southeast towards cooler, wetter cities in the southeast. Early in the epidemic, NHP cases were similarly identified in the hot, dry northern part of the region, in or around municipalities with the earliest human cases, and eventually progressing to the cooler, wetter south (Fig 2).

### Drought index

**Fig 3:**
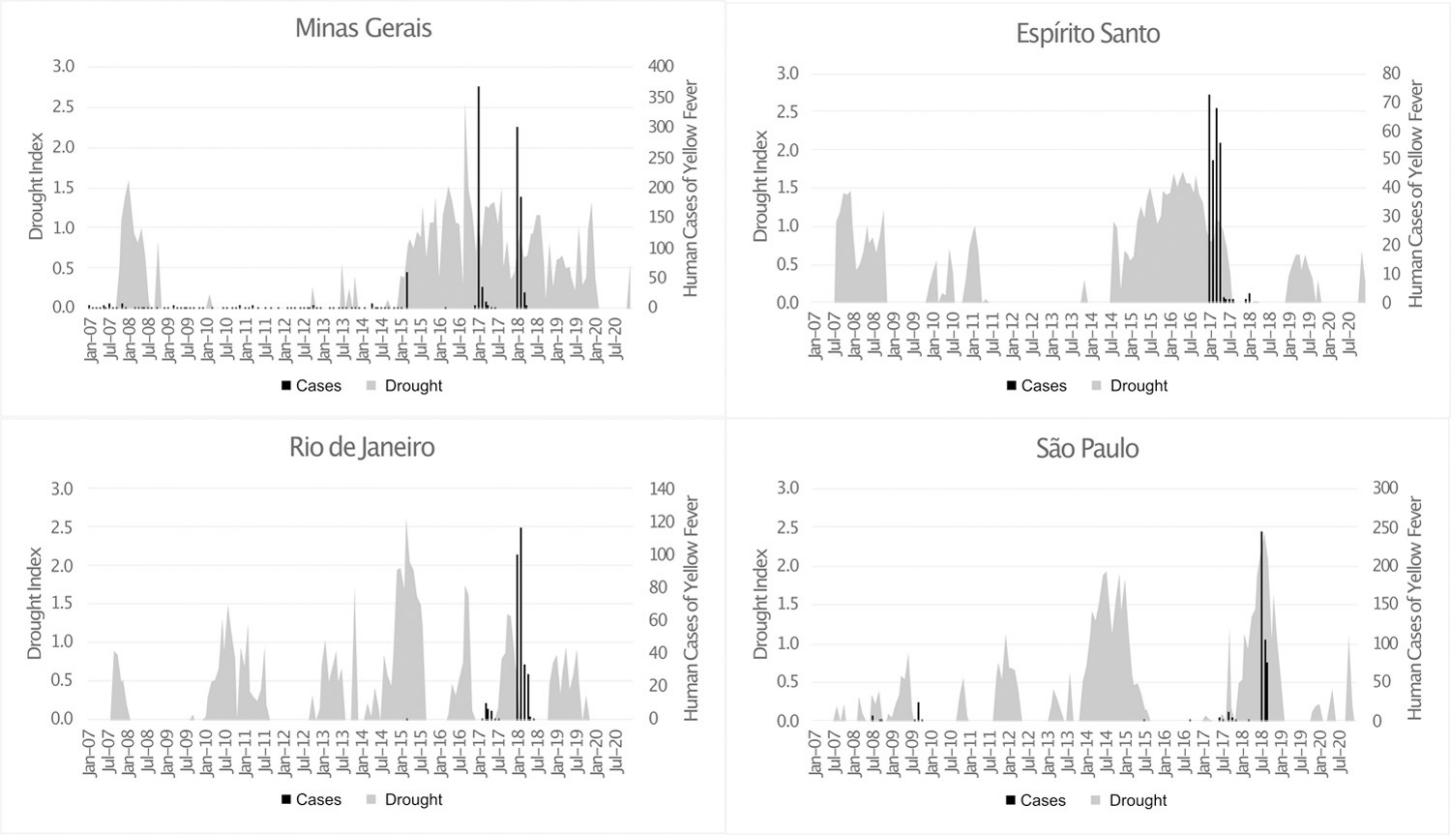
Timing of drought & yellow fever virus outbreaks. Abbreviations: Jan = January; Jul = July. The "Drought Index" is derived from the Standardized Precipitation Evapotranspiration Index (SPEI) which measures water balance based on rainfall and temperature. A negative value of SPEI indicates a water deficit; a negative SPEI is represented here by grey bars showing an increased drought index. The number of yellow fever virus cases reported in each state is represented by the black bars. Yellow fever virus outbreaks coincided with severe drought conditions in each of the four southeastern states of Brazil.

A major drought hit southeastern Brazil starting around 2012 and ending around 2019. We plotted a drought index as measured by the Standardized Precipitation Evaporation Index (SPEI) from January 1, 2007 to December 31, 2020 against confirmed YFV cases in each of the four southeastern states of Brazil (Fig 3).

The peak of the YFV epidemic coincided with increased drought in all four southeastern states. The drought index, SPEI, was consistently less than -1.0 (moderate drought) for a period of two to three years in both Minas Gerais and Espírito Santo when the epidemic hit those states in 2016. Furthermore, at the end of 2016 in Minas Gerais when and where this epidemic originated, the SPEI depression exceeded -2.5, well beyond the threshold of -2.0 to classify as extreme drought. The SPEI remained highly negative in Minas Gerais through 2018, coinciding with a second wave of the epidemic in that state. In contrast, the epidemic in Espírito Santo spiked in 2017 after three years of drought; it then was almost entirely spared from a second wave of cases in 2018 at the same time that their SPEI returned to zero (normal).

Rio de Janeiro and São Paulo, which were most affected by the second wave of the epidemic in 2018, had shorter and more intermittent spikes in the drought index in the years leading up to the epidemic, rather than the multiple years of sustained negative SPEI experienced by the more northern states of Minas Gerais and Espírito Santo. Notably, the SPEI in 2016 and the first half of 2017 (during the first wave of the epidemic further north) for both Rio de Janiero and São Paulo was near zero and then decreased to nearly -2.0 (extreme drought) in early 2018, again coinciding with the peak of the epidemic in those states.

The model fit between monthly cases and monthly SPEI as measured by Pearson’s correlation coefficient for Minas Gerais, Espírito Santo, Rio de Janiero, and São Paulo were 0.17, 0.16, 0.11, and 0.28, respectively. When limiting this analysis to peak summer months (December to April), when YFV transmission occurs, Pearson’s correlation coefficients mildly increased to 0.26, 0.27, 0.14, and 0.16. This relatively low model fit is driven largely by the several months of drought in which there were no cases of YFV.

### Rural /Outdoor exposure

Rural and outdoor exposure was assessed by demographic characteristics including sex, age, recent travel history, and occupation. In both univariate and multivariable analyses, confirmed cases of YFV were significantly more likely to be male (multivariable Odds Ratio (OR): 2.6; 95% Confidence Interval (CI): 2.2–3.0) and be of working age (multivariable OR: 1.8; 95% CI: 1.5–2.1) compared to discarded cases (Table 1). A recent history of any travel was also significantly associated with confirmed cases of YFV (multivariable OR: 2.8; 95% CI: 2.3–3.3).

**Table 1:** Demographic characteristics of confirmed versus discarded YFV cases in 2017 & 2018.

	Confirmed Cases (n = 2,097) N (%)	Discarded Cases (n = 6,999) N (%)	Univariate Analysis Odds Ratio (95% CI)	Multivariable Analysis Odds Ratio (95% CI)
Male	1,726 (82.3%)	4,183 (63.4%)	**2.6 (2.3–3.0)**	**2.6 (2.2–3.0)**
Working age (16–65 years old)	1,848 (88.1%)	5,374 (78.3%)	**2.1 (1.8–2.4)**	**1.8 (1.5–2.1)**
Unvaccinated	1,671 (86.2%)	2,818 (48.3%)	**6.7 (5.8–7.7)**	**6.4 (5.5–7.4)**
*Travel (Origin -> Destination)*				
Urban -> rural	112 (5.6%)	85 (1.3%)	**4.9 (3.7–6.5)**	**2.9 (2.1–4.1)**
Urban -> urban	220 (11.0%)	212 (3.3%)	**3.9 (3.2–4.7)**	**3.2 (2.5–4.0)**
Rural -> rural	29 (1.5%)	33 (0.5%)	**3.3 (2.0–5.4)**	**2.3 (1.3–4.2)**
Rural -> urban	21 (1.1%)	46 (0.7%)	1.7 (1.0–2.8)	1.3 (0.7–2.3)
None (reference)	1,623 (81.0%)	6,023 (94.1%)	**-**	**-**

Abbreviations: YFV = yellow fever virus; CI = confidence interval. Confirmed YFV cases were more likely to be male, working age, unvaccinated, and have a history of travel compared to discarded cases. Multivariable analysis also controlled for month and year. Bold font indicates result is statistically significant at the level of p<0.001. Cases were classified as confirmed or discarded by the Brazilian Ministry of Health based on laboratory and clinical characteristics; cases classified as indetermined were excluded from this analysis. Working age range was defined based on the Brazilian government’s minimum legal age of entry into the labor market and retirement age. Missing data: Age data was missing for 136 discarded cases and 0 confirmed cases. Vaccination status was missing from 1,163 discarded cases and 158 confirmed cases. Travel data was missing from 600 discarded cases and 92 confirmed cases.

**Fig 4:**
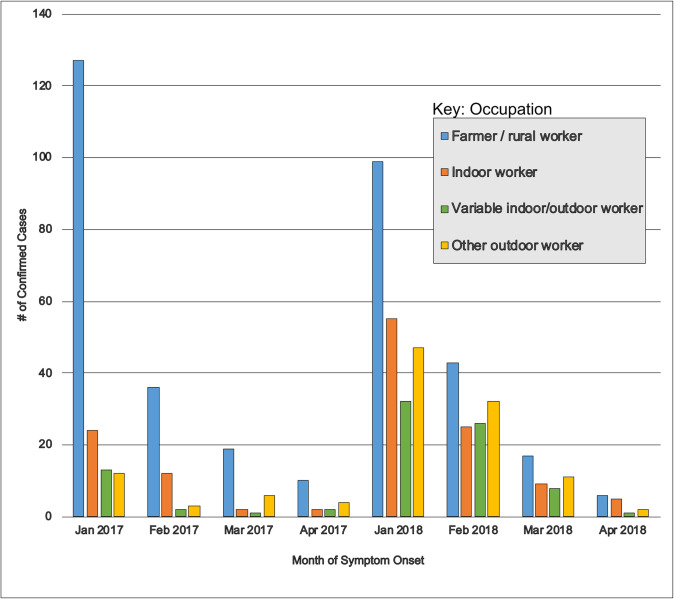
Occupation of yellow fever virus cases over the course of the epidemic. Abbreviations: Jan = January; Feb = February; Mar = March; Apr = April. Farmers and rural workers (blue bars) dominated the cases in the first year of the epidemic (2017). This was also the predominant group at the start of each wave of the epidemic (January 2017 & January 2018).

Occupation was available for 765 (36.5%) of the 2,097 confirmed cases in 2017 and 2018. Farmers/rural workers made up the largest occupational category with 398 (52.0%) of the 766 cases. Confirmed cases were more likely to be a farmer/rural worker than another occupation in 2017 compared to 2018 (OR: 3.16; 95%CI: 2.32–4.32). Similarly, confirmed cases were more likely to be a farmer/rural worker in January (i.e. the start each wave of the epidemic) compared to other months (OR: 1.81; 95%CI: 1.34–2.45). (Fig 4)

### Mechanistic Framework for the Role of Drought in Brazil’s YFV Outbreak

**Fig 5:**
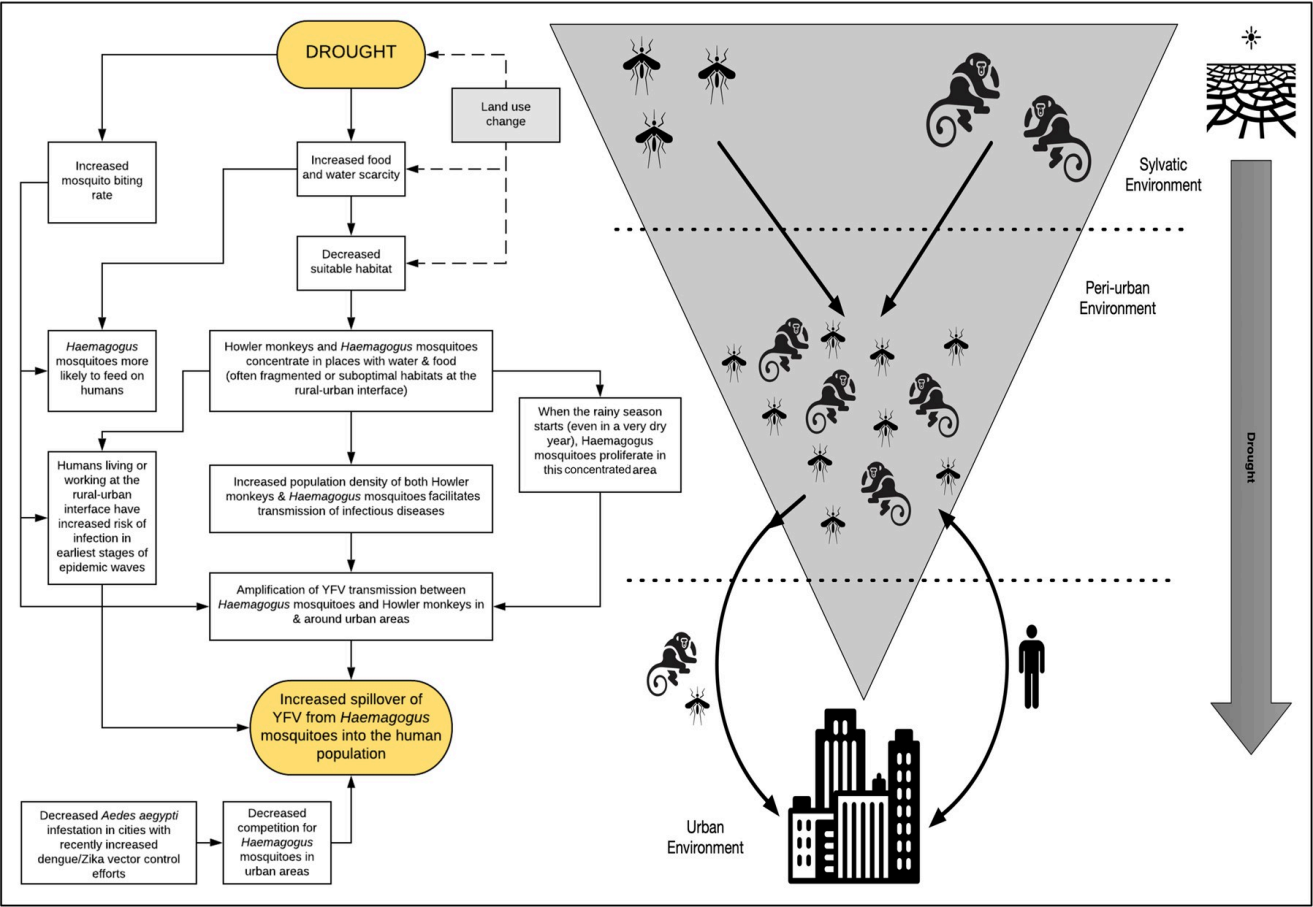
"*Mono-DrY*": *M*echanistic framework for the role of *Dr*ought in Brazil’s *Y*FV outbreak. Abbreviations: YFV = yellow fever virus. We provide a biologically plausible mechanism by which drought could have brought *Haemagogus* (forest) mosquitoes, howler monkeys, and humans into closer contact at the rural-urban interface promoting the amplification of YFV transmission and triggering the largest epidemic of YFV in southeastern Brazil in nearly a century. In addition to drought, this model considers two additional contributing factors: the ecology of *Aedes aegypti* mosquitoes as well as land use change. The land use change text box is highlighted in grey as further research is needed to explore the interaction between land use change and drought in triggering this epidemic.

We created a framework, which we have termed “Mono-DrY”, to explain how an extreme drought in SE Brazil could have sparked the spread of YFV via *Haemagogus* mosquitoes (Fig 5). In this model, environmental stress caused by drought decreases available habitat, forcing mosquitoes and NHPs to congregate in high densities in the few remaining areas with adequate food and water, particularly near rural-urban boundaries. Drought also increases mosquito biting rate which promotes viral transmission. Drought thereby results in amplification of transmission at the rural-urban interface and spilling over first into individuals with rural/outdoor exposure. Additional factors that could further contribute to this causal framework include land use change and decreases in urban *Aedes ageypti* populations.

This framework is biologically plausible and is based on well-established mosquito and non-human primate biology and behavior. YFV is transmitted in Brazil by *Haemagogus* mosquitoes which predominantly breed in pools of water in trees and feed on NHPs at the top of canopies, occasionally descending to bite humans when food is scarce. *Haemagogus* sp. can also travel long distances, up to 11.5 km in just a couple days [30]. During a drought, *Haemagogus* sp. could therefore seek out more optimal feeding and breeding grounds, concentrating around remaining water, NHPs, and humans. Howler monkeys frequently spend their days eating in the tops of canopies where *Haemagogus* sp. live [31]. Howler monkeys (*Alouatta sp*.) are highly susceptible to YFV; during NHP surveillance conducted during this epidemic, Howler monkeys were also the most common NHPs found to be infected with YFV and infected Howler monkeys were found in both rural and urban areas surveyed [2,24]. Marmosets (*Callithrix sp*.) were the second most common NHPs found to be infected, also in both rural and urban areas. Both Howler monkeys and marmosets are highly adaptable to fragmented, suboptimal habitats, particularly during periods of scarcity and at the rural-urban interface [31–34]. Furthermore, when forced into smaller patches, NHP population density increases and parasite load increases [35]. These NHPs commonly cross between rural-urban boundaries and were the two predominant NHP genera identified in the epizootic YFV surveillance program [1,2]. If NHPs and *Haemagogus* sp. were forced into higher density conditions in search of water and food, particularly near rural-urban borders, this could allow for amplification of YFV transmission once the hot rainy summer season started. Other studies have shown that increased habitat stress and fragmentation promotes increased density of both *Haemagogus* sp. and Howler monkeys [6,35]. There is even some evidence that mosquitoes may increase their biting rate during periods of drought due to dehydration, thereby further promoting viral transmission [36].

An additional factor that could have facilitated the spread of YFV from rural into urban areas at this time is open mosquito niches in urban areas. *Aedes aegypti* is typically the dominant mosquito species in urban centers in Brazil. However, severe outbreaks of dengue, chikungunya, and Zika virus in the years just preceding the yellow fever epidemic prompted aggressive mosquito control efforts. The *Aedes aegypti* index as measured in Rio de Janeiro at the time of this epidemic had decreased 2 to 3 fold at the start of the YFV outbreak compared to the previous decade [37]. Such a decrease in *Aedes aegypti* numbers could have allowed the typically forest dwelling *Haemagogus* sp. to take up residence in this open niche in urban centers. Although competition between *Aedes aegypti* and *Haemagogus* sp. has not been studied in this setting, such habitat competition between mosquitoes has been documented in prior studies [38,39]. This is further supported by several studies during this epidemic, which found no evidence of YFV transmission by *Aedes aegypti*, despite them being competent vectors in laboratory studies, but rather transmission in urban areas was predominantly by *Haemagogus* sp. and very rarely by other (non-aegypti) *Aedes* species [7,8]. This framework also highlights land use change, a factor associated with YFV outbreak risk in prior studies, which would also contribute to suitable habitat availability and requires further investigation [40,41].

## Discussion

Our results show that the YFV outbreaks of 2017–2018 coincided with periods of drought in all four southeastern states of Brazil affected by this unusual epidemic. We also trace the epidemic as it starts in the hot, dry northern region of Minas Gerais and spreads east and south into cooler, damper, and more urbanized areas. The pattern of this spatiotemporal progression is seen with both confirmed human and NHP cases. Lastly, we found an increased risk of YFV infection associated with demographic characteristics suggestive of outdoor and rural exposure, including male sex, working age, and recent travel. Additionally, we found that farmers/rural workers were the population first infected during this epidemic overall and at the start of each wave. Finally, we created a novel mechanistic framework to explain how drought could have sparked the spread of this epidemic, linking our new analyses with prior work on mosquito and non-human primate biology.

Our findings on the origin and spread of the epidemic are consistent with small genomic sequencing studies that suggest multiple introductions of YFV from Minas Gerais into the more urban states of Espírito Santo, Rio de Janeiro, and São Paulo [42–44]. This supports our hypothesis that this epidemic started in rural areas of Minas Gerais where intermittent transmission had previously occurred and that environmental conditions during this time were uniquely primed for YFV spread to neighboring states and urban centers where huge outbreaks had unfolded. This is consistent with other studies that demonstrated an overall southeastern spread of YFV through Brazil over the past two decades and during this epidemic in particular [20].

This 2017–2018 YFV outbreak occurred in a part of Brazil that had not experienced YFV transmission in nearly a century. Around the same time, Brazil experienced one of the most extreme droughts in the last century [18]. Furthermore, in the four states where these highly unusual YFV outbreaks occurred, we found that these YF events in each individual state coincided with peaks in the SPEI drought index over the last two decades. We noted that the outbreaks started in northern Minas Gerais which is the hottest and driest part of the southeastern region of Brazil and where the SPEI index reached a level of extreme drought just as the outbreak started. Our findings are further corroborated by a study conducted by Brazil’s National Center for Monitoring and Early Warning of Natural Disasters which demonstrated that the northern region of Minas Gerais was the area most severely affected by drought in 2016/2017 with an Integrated Drought Index of extreme drought [18]. Our findings echo other studies which implicated extreme weather events as playing a role in triggering anomalous YFV outbreaks in Africa [45,46].

This study had some limitations, most notably due to the episodic nature of this outbreak. The 2017–2018 YFV epidemic in southeastern Brazil was a single epidemic in a new geographic area, which makes it an important and interesting case study. Without a longer historical record of repeated epidemics in this region, the statistical analyses evaluating the relationship between this single epidemic and a severe drought is complex. Yet, it is highly suspicious that the reemergence of YFV in this area for the first time since the 1940s occurred at the same time as the worst drought this area experienced since the 1940s [3,17]. We demonstrated that the epidemic waves in each state overlapped with the periods of drought. Moreover, Minas Gerais, where the epidemic originated, was in an extreme drought leading into the start of the epidemic. Although we cannot attach statistical significance to this finding, these environmental observations in concert with our proposed biological mechanism (by which drought could have amplified YFV transmission at the rural-urban interface in Minas Gerais) supports our hypothesis that an extreme drought could have triggered this unusual epidemic.

This study was also limited by the data available from surveillance systems, which primarily captured hospitalized cases of YFV infection and would have missed mild or asymptomatic cases. Surveillance and hospital systems are also not necessarily equally distributed throughout this region and were likely most robust in urban areas, which could somewhat bias the distribution of cases to these areas. The non-human primate cases were also captured using a convenience sampling of NHP carcasses that were tested for evidence of YFV infection; this sampling method would also likely bias towards a more urban distribution. Even with this potential urban bias for both human and NHP distribution of YF cases, the epidemic clearly started in more rural areas and there was consistent evidence of increased risk with rural / outdoor exposure. Within the cases captured by the surveillance system, there was also some missing data. Although age and sex were nearly universally recorded, occupation was only available for about a third of confirmed cases. Detailed information about outdoor exposure for each occupation type was not available.

The spread of YFV into areas that had been YFV free for nearly a century signals changing conditions. Our study identifies patterns which suggest increased stress during a severe drought promoted the amplification of YFV transmission between mosquitoes and NHPs at rural-urban boundaries, ultimately spilling over into a large human epidemic that spread from rural to urban areas. In this study we examined temperature, precipitation, and evapotranspiration as components of drought, a climate variable that has not previously been evaluated in this YFV epidemic. Temperature, rainfall, and humidity also play important roles in mosquito life cycles [47,48]. The relationship between these climate variables and YFV transmission have previously been examined using mechanistic, machine learning, and statistical models [19,22]. Considering our findings, we argue that drought, particularly extreme drought, is another mechanism by which temperature and precipitation can affect YFV transmission. More work is needed to incorporate drought into our mechanistic framework for modeling arbovirus transmission. Because drought can be defined and measured in numerous ways and have multiple downstream effects on mosquitoes, animal and human hosts, more work is also needed to evaluate how best to model drought for arbovirus transmission models. Given that temperature and rainfall are already part of many arbovirus models and have good spatial resolution globally, the SPEI is a natural starting point, but other measures could also be considered.

We propose that drought contributed to resource scarcity and habitat degradation, therein creating increased mosquito and NHP density. Land use change also contributes to habitat fragmentation and has been independently linked with YFV dispersion in prior studies [40,41]. Other models have demonstrated that a combination of climate variables, human footprint, and population density, all increased model fit in a machine learning model of this epidemic [19]. More research is needed to explore how the interaction between land use change and drought may have altered mosquito and NHP behavior. The reemergence of YFV in densely populated parts of Brazil raises serious concerns about the potential for the reestablishment of an urban YFV transmission cycle which could involve *Aedes aegypti* in the future. More research is also needed to understand what factors may have prevented YFV from reestablishing an infection cycle in *Aedes aegypti* during this epidemic and therefore what could be done to minimize this future risk.

In summary, we demonstrate how an unusual drought in this region could have amplified YFV transmission at the rural-urban interface and triggered this epidemic. YFV risk could increase further in southeastern Brazil, particularly in light of climate studies projecting that the São Francisco River Basin feeding this region is expected to experience increasing drought in the coming century [49,50]. Our “MonoDrY” framework also highlights the importance of further studying the relationship between drought and land use change on habitat fragmentation and YFV amplification. Policies regarding land use practices and water management could attenuate the impact of drought on YFV amplification. Another modifiable risk factor which can play a key role in the occurrence and size of YFV outbreaks is vaccination. Vaccination coverage was low at the start of this epidemic and the rapid expansion of vaccination coverage, predominantly through ring vaccination, was a key part of efforts to effectively control this outbreak [23,51]. We acknowledge that the formulation of vaccination policy is a complex process that must take into consideration a variety of factors, however, in light of the potential for sylvatic YF epidemics to expand over considerable distances, decision makers should evaluate immunization policies including mass vaccination strategies [52]. A combination of factors contributes to YFV transmission. In this study we highlight the previously unrecognized role that drought could have played in triggering this unusual epidemic. Reconstructing the relationship between drought and YFV transmission in southeastern Brazil creates an opportunity to consider new interventions and policies to mitigate the risk of future outbreaks.
